# Practice changes beta power at rest and its modulation during movement in healthy subjects but not in patients with Parkinson's disease

**DOI:** 10.1002/brb3.374

**Published:** 2015-09-23

**Authors:** Clara Moisello, Daniella Blanco, Jing Lin, Priya Panday, Simon P. Kelly, Angelo Quartarone, Alessandro Di Rocco, Chiara Cirelli, Giulio Tononi, M. Felice Ghilardi

**Affiliations:** ^1^ Department of Physiology, Pharmacology and NeuroscienceCUNY Medical SchoolNew YorkNew York10031; ^2^ Department of Biomedical EngineeringCCNYNew YorkNew York10031; ^3^ Department of Neurosciences, Psychiatry and Anaesthesiological SciencesUniversity of MessinaMessina98125Italy; ^4^ The Fresco Institute for Parkinson's and Movement DisordersNYU‐Langone School of MedicineNew YorkNew York10016; ^5^ Department of PsychiatryUniversity of MadisonMadisonWisconsin53719

**Keywords:** Event‐related desynchronization, event‐related synchronization, kinematics, motor task, plasticity, RRID:nif‐0000‐00076, RRID:nlx_143928, RRID:nlx_155825, RRID:rid_000042

## Abstract

**Background:**

PD (Parkinson's disease) is characterized by impairments in cortical plasticity, in beta frequency at rest and in beta power modulation during movement (i.e., event‐related ERS [synchronization] and ERD [desynchronization]). Recent results with experimental protocols inducing long‐term potentiation in healthy subjects suggest that cortical plasticity phenomena might be reflected by changes of beta power recorded with EEG during rest. Here, we determined whether motor practice produces changes in beta power at rest and during movements in both healthy subjects and patients with PD. We hypothesized that such changes would be reduced in PD.

**Methods:**

We thus recorded EEG in patients with PD and age‐matched controls before, during and after a 40‐minute reaching task. We determined posttask changes of beta power at rest and assessed the progressive changes of beta ERD and ERS during the task over frontal and sensorimotor regions.

**Results:**

We found that beta ERS and ERD changed significantly with practice in controls but not in PD. In PD compared to controls, beta power at rest was greater over frontal sensors but posttask changes, like those during movements, were far less evident. In both groups, kinematic characteristics improved with practice; however, there was no correlation between such improvements and the changes in beta power.

**Conclusions:**

We conclude that prolonged practice in a motor task produces use‐dependent modifications that are reflected in changes of beta power at rest and during movement. In PD, such changes are significantly reduced; such a reduction might represent, at least partially, impairment of cortical plasticity.

## Introduction

Recent evidence from our laboratory indicates that performance of a specific learning task induces local changes in the oscillatory EEG activity not just during the task itself, but also in spontaneous recordings during the resting state. These performance “signatures” have been documented for 24‐h training with driving simulation or listening to audiobooks (Hung et al. 2013) as well as for shorter periods of about 40 minutes involving visuo‐motor adaptation to rotated displays (Landsness et al. 2011) and learning of visual sequences (Moisello et al. 2013). In particular, we found that, after a 40‐minute visual learning, the EEG changes at rest were confined to the regions active during learning and were task‐specific, as they did not occur after performance of the same duration with other tasks. Interestingly, these EEG changes were not proportional to either the learning rate or the level of knowledge achieved during the performance. For these reasons, they were interpreted as reflecting not the learning per se but rather the intensive use of these areas. In other words, they were interpreted as signs of use‐dependent plasticity, possibly independent from learning outcomes (Moisello et al. 2013).

It is not known whether similar local EEG changes are present after tasks without overt learning or fatigue. Evidence that this might be the case comes from our recent study with TMS (transcranial magnetic stimulation), where we found a decrease in motor cortex excitability after ten minutes of repetitive finger movements, a task with negligible learning and without signs of neuromuscular fatigue (Crupi et al. 2013). We concluded that the significant decrease in cortical excitability after that simple exercise, again, reflected processes related to use‐dependent plasticity.

In general, the studies to define use‐dependent plasticity in motor areas have been usually based on measures of cortical excitability collected with TMS most commonly after an experimental stimulation or, in fewer cases, after a task. However, such plasticity‐related phenomena have been rarely examined in terms of oscillatory activity, despite the well‐known presence of spontaneous oscillations in the beta band (15–30 Hz) over the sensorimotor and other areas. Only recently, some studies in healthy subjects have shown that theta burst TMS protocols, which modulate local plasticity (Huang et al. 2005), induced local changes not only in cortical excitability but also in beta power (Hsu et al. 2011; Noh et al. 2012; McAllister et al. 2013). Interestingly, one of these studies (McAllister et al. 2013) showed that some of the subjects did not respond to these TMS protocols with the characteristic changes in cortical excitability; these *nonresponders* also lacked of the characteristic beta power increase that was present after TMS in all the *responders*. As also suggested by these authors (McAllister et al. 2013), this finding is of particular relevance to understand the motor impairment typical of PD (Parkinson's disease), a disorder characterized by impaired plasticity and alterations in the beta frequency range. In fact, on one hand, reduced responses to LTP (long‐term potentiation)‐like protocols and decreased retention of newly learned skills have been shown in PD (Morgante et al. 2006; Ueki et al. 2006; Marinelli et al. 2009; Bedard and Sanes 2011; Kishore et al. 2012; Moisello et al. 2015). On the other hand, many electrophysiological studies, with techniques ranging from recordings of local field potentials to MEG (magneto‐encephalography), have now consistently demonstrated a pathologically elevated background of beta power in the spontaneous, resting state EEG that is partially linked to disease duration and bradykinesia and that can respond to levodopa treatment and deep brain stimulation (Brown and Marsden 1999; Levy et al. 2002; Priori et al. 2004; Sharott et al. 2005; Hammond et al. 2007; Mallet et al. 2008; Giannicola et al. 2010; Pollok et al. 2012; Tan et al. 2013).

Besides abnormalities in the resting state EEG, recent studies have found that patients with PD might exhibit abnormal modulation of movement‐related beta oscillations (Heinrichs‐Graham et al. 2014). In healthy subjects, beta power over the sensorimotor areas starts decreasing before movement onset, reaches a negative peak during execution (the so‐called *event‐related desynchronization*, ERD) and increases after the movement (*event‐related synchronization*, ERS) (Pfurtscheller and Lopes da Silva 1999; Toma et al. 2002). In PD, movement‐related beta modulation is present albeit with a reduced amplitude (Delval et al. 2006; Lim et al. 2006; Degardin et al. 2009; Heinrichs‐Graham et al. 2014): this alteration could be related to the somatosensory abnormalities that are often present in PD (Conte et al. 2013). In fact, it has been suggested that beta ERD and ERS, respectively, reflect the attenuation and the reactivation of sensory afferences during motor performance, a phenomenon called “sensory gating”. It is not known whether, in either controls or PD, prolonged practice in a motor task produces changes of movement‐related beta modulation. Indeed, if beta activity reflects changes in cortical excitability and plasticity, as suggested by McAllister et al. (2013), one should expect, in healthy subjects, changes of beta activity not only with theta burst stimulation or similar TMS protocols but also with continuous training in a specific motor task.

In this study, we determined whether beta power at rest and its modulation during movement change with extended, repetitive motor practice in subjects with PD and age‐matched controls. We focused on the electrodes over the left and right sensorimotor and fronto‐central regions, three areas where beta ERD and ERS are clearly modulated during voluntary movements (see (Kilavik et al. 2013) and references therein). High‐density EEG was recorded during the 40‐minute performance of a motor task requiring reaching movements to targets presented in an unpredictable order (Ghilardi et al. 2000; Perfetti et al. 2011). In both groups, we first, defined the topography of beta oscillations during the task and determined whether beta ERD and ERS changed with practice. Then, we ascertained whether such motor training left a local trace in EEG at rest. We found that, in controls, beta power modulation changed with practice and that a posttask trace was present in the spontaneous EEG. However, all these changes were significantly reduced in patients with PD compared to controls.

## Methods

### Subjects

Fifteen patients with PD (three females, age: mean 60.7 ± SD 6.7 years, Hoehn & Yahr stage: 2.2 ± 0.4; disease duration: 6.7 ± 4.1 years; UPDRS [Unified Parkinson's Disease Rating Scale] – III [motor section] score: 19.1 ± 8.4; LED [Levodopa Equivalent Dosage]: 538.8 ± 268.9) and sixteen age‐matched controls with normal neurological examination (nine females, age: 60.1 ± 8.3 years) participated in this study. All subjects were right‐handed as determined by the Edinburgh inventory (Oldfield 1971) and had normal or corrected vision. Controls had no history of neurological or psychiatric disorders. Patients were tested in ON state, on their regular medication schedule. The experiments were conducted with the approval of our Institutional Review Board. Written informed consent was obtained from all participants.

### Experimental design

For all subjects, the experimental session started around 9 am. Subjects were outfitted a 256‐channel EEG cap (Electrical Geodesics Inc., Eugene, OR). Three minutes of RS (resting state) EEG were recorded before (RS1) and after (RS2) performance of a motor task. During RS‐EEG, subjects were asked to relax, to keep their eyes open and to fixate on a black circle (0.5 cm radius) in the center of a computer screen.

### Motor task

General features of the motor task have been detailed in previous studies (Ghilardi et al. 2000, 2003). Briefly, subjects moved a cursor on a digitizing tablet (sampling rate 200 Hz) with their right hand to targets presented on a screen, with a smaller circle indicating the cursor position. Targets were eight circles (1 cm radius) equidistant (4 cm) from the central starting point (indicated by a small cross) in the middle of the screen. The eight target circles and the position of the cursor on the screen were visible at all time. Upon presentation, one of the targets turned black for 400 msec. Targets blackened in random order, at 1.5 sec intervals. Subjects were instructed to make out‐and‐back reaching movements from the starting point to the presented target without corrections, as fast and accurately as possible, and to reverse sharply within the target without stopping. They were also asked to move as soon as possible, thus minimizing reaction time, but also to avoid anticipation or guessing. Subjects were trained to reach a hit rate of 95%. This was usually accomplished within ten minutes. Next, they performed a total of 840 movements in 10 blocks of 84 movements each. After each block, subjects paused for about a minute. Each session lasted approximately 40 min. As in previous publications (Ghilardi et al. 2000, 2003), we computed several spatial and temporal measures for each movement, including: reaction time, the time from the target appearance to the movement onset; amplitudes of peak velocity and peak acceleration; movement time, the time from movement onset to reversal; movement extent or amplitude as the vector length from onset to reversal point; directional error, the difference between the target direction and the direction of the movement at the instant of peak velocity. For each subject, we discarded from both kinematic and EEG analyses the movements that met one of the following criteria: movement or reaction time exceeding 2 SD from the subject's mean; movements directed to the wrong target (directional error >22°); previous movement ending 100 msec or less from the current target presentation. The average number of valid trials per block did not differ between groups and was 64.5 ± 6.4 in the controls and 61.2 ± 10.9 in patients with PD (unpaired *t*‐test: *P* = 0.29). For each kinematic variable, to verify the effect of PD and practice, we performed a mixed‐model ANOVA with group (PD, Controls) as between‐subject effect and practice (Block1 to Block10) as within‐subject effect.

### EEG recordings

EEG was recorded for the entire duration of the experimental session, both during the task performance and in the resting state periods. Data were collected at a sampling rate of 1000 Hz using the high impedance amplifier Net Amp 300 and Net Station 4.3 (Electrical Geodesics Inc.). Impedances were kept below 50 kΩ. From the original 256 electrodes, we removed 73 channels located on the cheeks and on the neck. The remaining 183 electrodes were used for further analysis. During the recording, the EEG signal was referenced to the Cz electrode. For analysis, data were down‐sampled to 250 Hz and rereferenced to the average across the 183 electrodes.

#### Preprocessing

We preprocessed the data with NetStation 4.3 software (Net Station EEG Software, RRID:nlx_155825, Electrical Geodesics Inc.) and the Matlab‐based public license toolboxes EEGLAB (RRID:nif‐0000‐00076, Delorme and Makeig 2004). Subsequent analysis also included functions from the Fieldtrip toolbox (RRID:nlx_143928, Oostenveld et al. 2011). Briefly, the continuous EEG signal was filtered between 0.5 and 80 Hz, with a notch filter at 60 Hz. Channels affected by bad scalp‐electrode contact were visually identified and replaced with spherical spline interpolation (number of bad channels, mean ± SD, Controls: 1.9 ± 1.8; PD: 1.3 ± 1.3). EEG recorded during the motor performance was segmented into 3‐sec epochs aligned with movement onset (−1 sec to +2sec). RS‐EEG was segmented into consecutive 2‐sec epochs. Epochs containing sporadic artifacts (abnormal tension bursts, cough, or similar) were rejected by visual inspection. Stereotypical artifacts, such as blinks, eye movements, and muscle tension, were removed by Independent Component Analysis (Makeig et al. 2004; Onton and Makeig 2006).

#### EEG analysis – motor task

**Figure 1:**
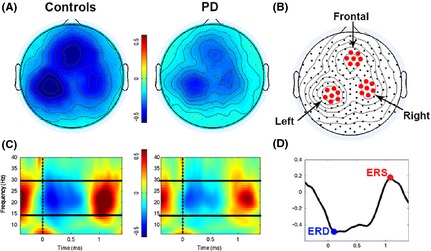
EEG data analysis. (A) Distribution of the mean beta power modulation depth (% change) as measured from maximal ERD (event‐related desynchronization) to maximal ERS (event‐related synchronization) in the control (Left) and PD (Parkinson's disease) (Right) groups. Topographies are averaged over all valid trials in each subject. Despite the overall smaller modulation in PD, in both maps it is possible to notice the presence of local maxima in three main areas. (B) Identification of ROIs (region of interests). The electrode with maximal modulation in each of the three aforementioned regions was selected together with the six immediate neighbors. (C) Time‐frequency plot for the event‐related spectral change over the Left ROI obtained by averaging all trials of the control (Left) and PD (Right) groups. On the *X*‐axis, 0 and the vertical dotted line indicate the time of the movement onset. The solid horizontal lines indicated the limits of the beta range. Notice that the strongest power modulation occurs over the beta range, with a uniform distribution centered around 22 Hz. (D) Representation of power variation in the beta band during a block of movements. The blue and red dots indicate the value of ERD and ERS, respectively.

After preprocessing, all artifact‐free trials from the motor task were submitted to time‐frequency and statistical analyses. For all channels, we computed time‐frequency representations in the range from 6 to 40 Hz using a short‐time Fourier transform approach (Hanning taper, time step‐size of 20 msec, 7 cycles adaptive window width, 1 Hz frequency step). For this study, we focused our attention on beta oscillations, that is, the range from 15 to 30 Hz. Indeed, this is the rhythm that undergoes the strongest and most consistent modulation during movement (see Fig. 1C and (Kilavik et al. 2013; Tan et al. 2014; te Woerd et al. 2014)). As the movement‐free time interval between consecutive movements is rather short, change in oscillatory power during movement was defined as percent change with respect to the resting state interval at the beginning of the session (RS1). As previously reported in numerous studies, beta power starts decreasing before movement onset, reaches a negative peak (ERD) during movement execution and finally shows a characteristic rebound (ERS) after the movement end (Fig. 1D). To identify the sensors showing the strongest beta modulation, we averaged the normalized beta band power for all valid trials and plotted the scalp distribution of the difference between maximal ERD and ERS in each group (Fig. 1A). The results show that the topography of beta modulation (which is similar in the two groups, despite being lower in patients, see Fig. 1A) is mostly focused over three areas involving left and right parietal electrodes as well as medial frontal electrodes. Therefore, for each of these three areas, we identified the electrode with the maximum beta modulation and included the six immediate neighbor electrodes to define the three ROI (region of interest), that is, the Left, the Right, and the Frontal ROIs (see Fig. 1B). The averaged beta power values over the seven electrodes in each of three ROIs for each Block was used to define the time course of beta modulation across the movements, with the focus on the computation of the minimum and the maximum peak values (ERD and ERS, respectively, Fig. 1D). Since beta ERD and ERS are very variable on a single trial level, we first obtained an average beta power for each block (i.e., up to 84 trials) and then we computed single maximum and minimum point on this average time course.

#### EEG analysis – resting state

Time‐frequency representations were extracted for each epoch of the RS for all electrodes, as described in the previous section for the motor task data. The resulting data were averaged over each time point and epoch in order to obtain an average beta power value for RS1 (before the motor task) and RS2 (after the motor task) for each subject. We then computed the change in resting power (RS change) for each subject as: [(RS2‐RS1)/RS1]%.

### Statistical analysis

All statistical analyses were conducted using SPSS v20 (RRID:rid_000042, SPSS Inc., Chicago, IL). To quantify the changes over the course of practice, for each ROI, we performed a mixed‐model ANOVA on ERD and ERS with Group (PD, Controls) as between‐subject effect and Practice (ten Blocks) as within‐subject effect. In case of significant effects, the differences between Block1 and Block10 were used for correlative analyses with resting state and performance measurements. To verify group differences in beta power, mean scalp RS beta power of controls and patients with PD were compared using unpaired *t*‐scores at the sensor level. RS changes were computed separately for each group, using a one‐sample *t*‐test to verify the statistical significance. We then plotted topographic maps of the *t*‐scores and corresponding probability, with different levels of correction for multiple comparisons. Unless otherwise stated, results were considered significant with a *P*‐value <0.05. In case of multiple comparisons, Bonferroni correction was applied.

## Results

### Motor performance changes with practice in PD and controls

**Figure 2:**
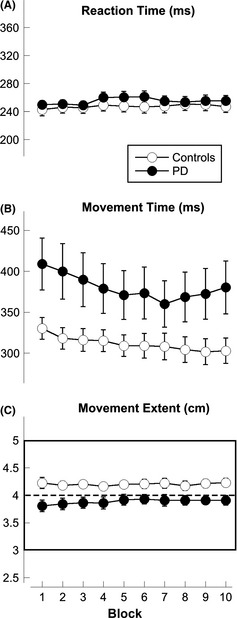
Kinematic results across each movement block. The circles represent the mean for the controls (empty circles) and the patients with Parkinson's disease (filled circles). The bars represent the standard errors. (A) Mean Reaction Time. (B) Mean Movement Time. (C) Mean Movement Extent. The rectangle in Figure 2C indicates the range of the target extent, whereas the dotted line in the middle indicates the center of the target.

All participants completed the 40‐minute session. In general, all movements were straight with overlapping out‐and‐back strokes and with bell‐shaped velocity profiles. As expected for ballistic movements, both peak velocity and peak acceleration correlated with movement time (*r* = 0.89 and 0.78, respectively, *P* < 0.0001). Since movement time, peak velocity, and acceleration were highly correlated and showed the same trend with practice, we will report only the results for movement time (see Fig. 2).

Mean reaction time was similar in controls and PD (*F*(1,29) = 0.51, *P* = 0.48) and increased by 4.8 (±2.8) ms in both groups from the beginning to the end of the session (*F*(9,261) = 2.87, *P* = 0.003, Fig. 2A). Average movement time was longer in PD than in controls (*F*(1,29) = 4.33, *P* = 0.046), but it decreased with practice in both groups (Practice: *F*(9,261) = 10.31, *P* < 0.001; Practice × Group: *F*(9,261) = 1.70, *P* = 0.09; Fig. 2B). Movement extent was also significantly different in the two groups, with PD performing shorter movements than controls (*F*(1,29) = 7.20, *P* = 0.01, see Fig. 2C). In both groups, movement extent did not change with practice (Practice: *F*(9,261) = 1.24, *P* = 0.27; Practice × Group *F*(9,261) = 1.07, *P* = 0.38).

### Modulation of movement‐related Beta oscillations changes with practice

**Figure 3:**
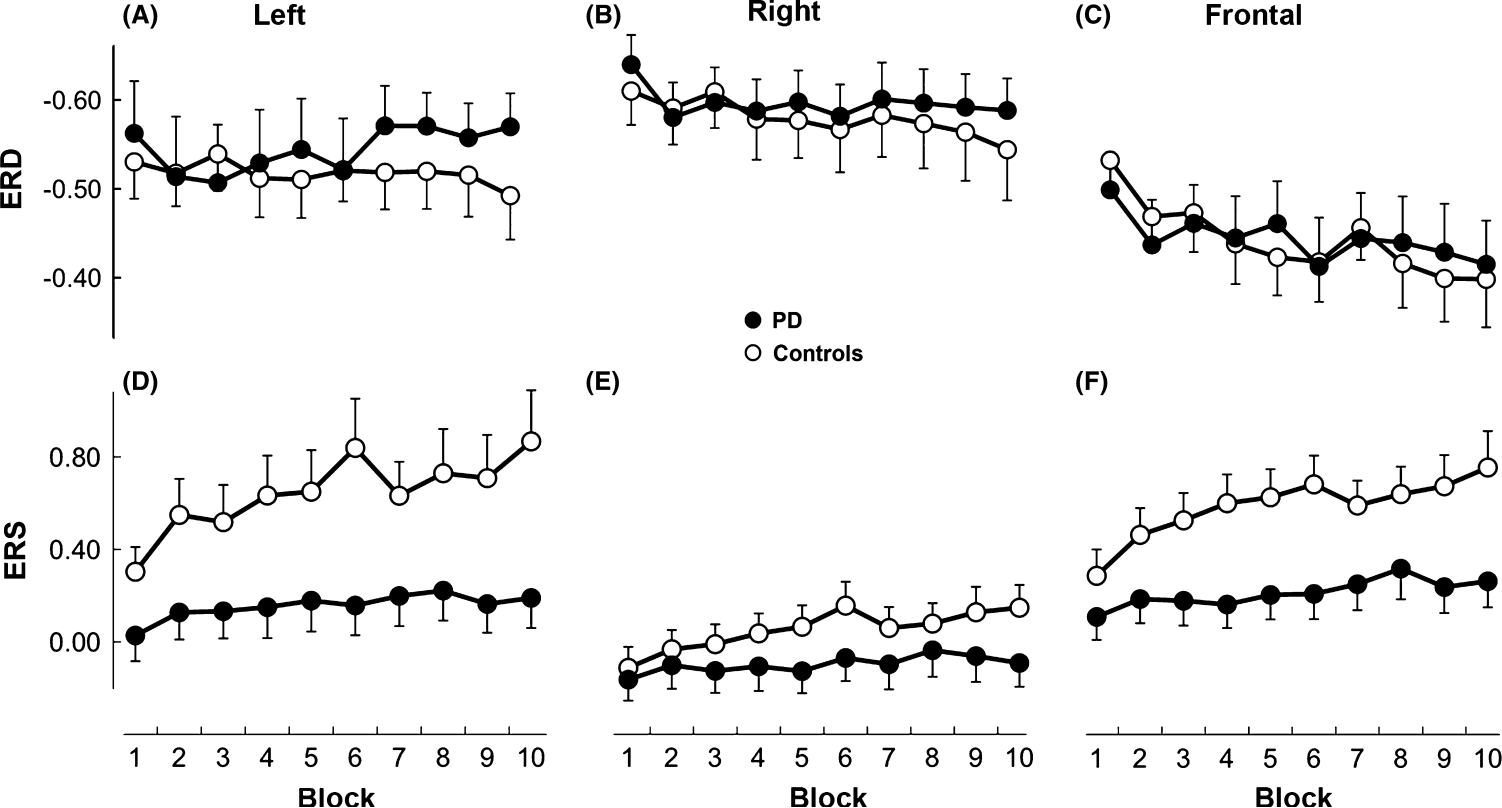
ERD (Event‐related desynchronization) and ERS (event‐related synchronization) changes during practice. For each of the three ROIs (region of interests), mean normalized ERD and ERS are plotted for each block in the Patient and Control groups. Bars represent standard errors. Please note that the scale of all ERD graphs (first row) is plotted in reverse to facilitate the interpretation, as greater ERD values correspond to more “negative” values. Also note that power is expressed as relative change from the initial resting state power (RS1), that is, a value of “+0.40” equals a 40% increase compared to the resting state. Negative values indicate power values lower than the resting state.

**Figure 4:**
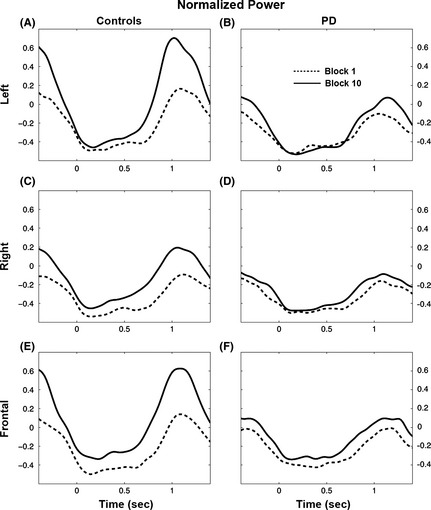
Beta power in the first and last Blocks. Average beta power during Block1 (dotted lines) and Block10 (solid lines) in the Control (Left column) and Parkinson's disease (Right column) in the Left (A, B), Right (C, D) and Frontal (E, F) region of interests. On the *X*‐axis, 0 indicates the time of the movement onset.

As detailed in the Methods, we analyzed the changes of beta ERD and ERS during practice in the three ROIs depicted in Figure 1B. The quantitative results are illustrated in Figures 3 and 4, whereas the results of the ANOVAs assessing the effects of practice, group and their interaction are reported in Table 1. The results of post hoc tests are described in the main text.

**Table 1:** Results of mixed‐model ANOVA on beta ERD and ERS for each ROI

	df	Left ERD		Right ERD		Frontal ERD	
		*F*‐value	*P*‐value	*F*‐value	*P*‐value	*F*‐value	*P*‐value
Group	1	0.18	0.67	0.09	0.77	0.001	0.97
Practice	9	0.63	0.77	**2.85**	**0.003**	**8.58**	**<0.00001**
Practice × Group	9	1.20	0.30	0.83	0.59	1.21	0.29

	df	Left ERS		Right ERS		Frontal ERS	
		*F*‐value	*P*‐value	*F*‐value	*P*‐value	*F*‐value	*P*‐value
Group	1	**5.59**	**0.02**	1.26	0.27	**5.79**	**0.02**
Practice	9	**7.24**	**<0.00001**	**7.30**	**<0.00001**	**7.51**	**<0.00001**
Practice × Group	9	**2.60**	**0.007**	**2.23**	**0.02**	**2.22**	**0.02**

ERD, event‐related desynchronization; ERS, event‐related synchronization; ROI, region of interest. Significant values are highlighted in bold.

#### Left ROI

Beta ERD was similar in PD and controls and did not change with practice in both groups (Fig. 3A,D). Beta ERS, on the other hand, was significantly greater in controls. In the control group, it increased significantly with practice so that, by Block4, it was significantly different from Block1 (*P* always <0.004 in all post hoc tests comparing Block1 to Block4 and later Blocks, Fig. 3D). No significant effects of practice were found for the PD group (*P* always >0.8). Inspection of the average beta power (Fig. 4A,B) confirmed these results, with a substantial increase in ERS during Block10 compared to Block1 in the Control group and a lack of such effect in the PD group.

#### Right ROI

Beta ERD values were similar in the two groups and decreased significantly (i.e., power became less negative) in the course of practice (Fig. 3C,D). However, such decrease was not significant when analyzing the data of the two groups separately with post hoc tests (*P* always >0.12). Inspection of the average of beta power in the first and last block (Fig. 4C,D) confirmed that ERD changes were rather small in both groups. On the other hand, beta ERS increased significantly only in the control group: post hoc tests revealed differences from Block1 starting at Block5 (*P* always <0.008). Practice did not affect beta ERS of the PD group (*P* always>0.7).

#### Frontal ROI

Beta ERD values, which were similar in PD and controls, decreased significantly with practice in both groups (Fig. 3E,F). In fact, post hoc tests revealed that, in controls, ERD values starting in Block4 were significantly lower than in Block1 (*P* < 0.003); in the PD group, this happened in Block6 and 10 (*P* < 0.03). In the control group, beta ERS values were on average greater than in PD and increased significantly with practice: starting from Block4, ERS values were statistically different from those of Block1 (*P* < 0.0007). No significant practice‐related changes were found in the PD group (*P* always>0.6).

We then asked whether the practice‐related changes in beta modulation were reflected in the changes in performance indices or clinical characteristics of PD. Thus, we first computed Pearson correlation coefficients between the change in ERD and ERS (differences between Block1 and Block10) and the corresponding change in kinematic and clinical indices. No significant linear correlations were found in either combined or single group analyses. Nevertheless, we further explored the hypothesis that the changes in ERS/ERD from Block1 to Block10 were due to the fact that movements in Block10 were significantly faster than those in Block1. Thus, for each subject, we selected 20 movements from Block1 and 20 from Block10 with comparable movement time (mean ± SE of selected movements: Block1: 303.4 ± 15.1 msec; Block10: 302.2 ± 15.6 msec, paired *t*‐test *P* = 0.5). We reasoned that, if the difference in ERS were due to movement time changes, we would expect similar values of ERS in these two groups of movements. However, the selected movements in Block10 showed still significantly greater ERS values (1.62 ± 1.10 *μ*V^2^/Hz) than the movements of comparable speed in Block1 (1.40 ± 1.14 *μ*V^2^/Hz, paired *t*‐test *P* < 0.05). This result suggests that the ERS changes observed with practice cannot be a mere effect of decreased movement duration.

In summary, we found that practice consistently affected the amplitude of the ERS over all the three ROIs and somewhat the amplitude of the ERD over the Right and Frontal ROI. As shown in Figure 4, these effects were more evident in the controls than in the patient group.

### Resting state beta power increases after practice

**Figure 5:**
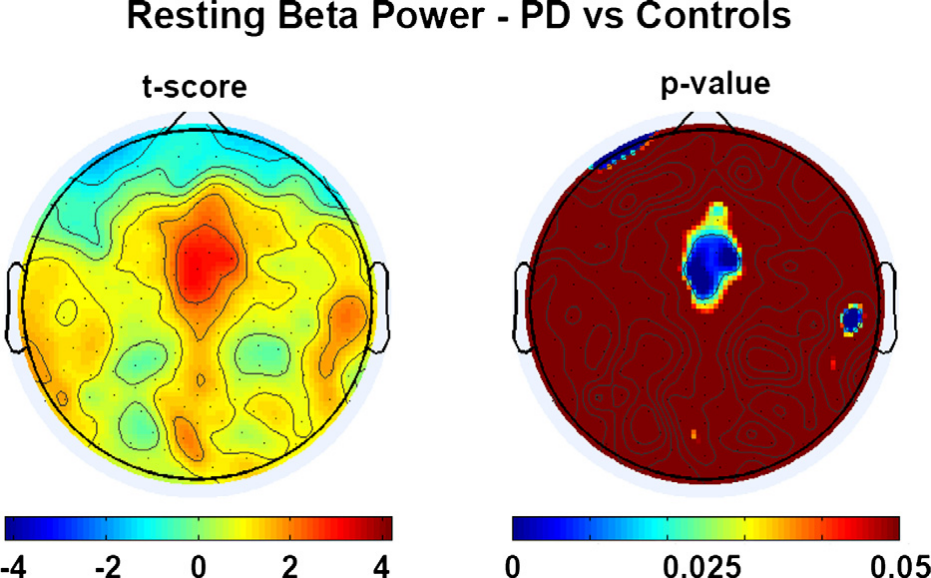
RS (Resting state) Power differences between groups. Topographic distribution of the *t*‐scores and associated probability for the comparison between beta power at rest in controls and patients. These results were obtained averaging RS1 and RS2 data; however, virtually identical distributions were obtained when computing data from the two RS intervals separately. The comparison was performed after averaging RS1 and RS2 in each group.

As shown in Figure 5, a group comparison of the average scalp maps of beta power during rest showed that beta power was significantly greater in patients with PD, especially in the electrodes over a medio‐frontal area (Fig. 5B, *P* < 0.005). The values of beta power at rest in these electrodes significantly correlated with the duration of PD (*r* = 0.64, *P* < 0.005). These results were obtained averaging RS1 and RS2 data; however, virtually identical distributions were obtained when computing data from the two RS intervals separately. The comparison was performed after averaging RS1 and RS2 in each group.

**Figure 6:**
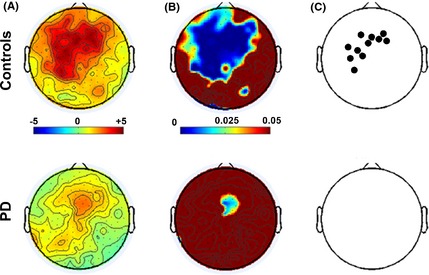
Changes in Resting state EEG after 40‐min motor task. (A) Scalp distribution of one sample *t*‐statistics for the percentage resting state increase, in Controls and patients with Parkinson's disease. (B) Uncorrected probability level (*P*‐value) associated with the *t*‐statistics. (C) Black dots indicate the electrodes that survived the correction for multiple comparisons over the 183 electrodes (*P* < 0.00027).

We then explored the practice‐related changes of beta power at rest in the two groups separately. In each subject, we computed for each electrode on the scalp the difference between the beta power during the resting state before and that after the motor task, in percentage, as outlined in the Methods. In the control group, the maps of *t*‐scores and associated probability values (Fig. 6A,B) showed a highly significant increase in beta power that was stronger on the electrodes over frontal and left areas. The *t*‐score map of the patient group displayed only a weak power increase in electrodes over the medio‐frontal area (Fig. 6A,B) that did not survive when correction for multiple comparisons over the 183 electrodes was used (*P* < 0.00027, Fig. 6C). Conversely, the use of such a correction did not substantially change the results in the control group (Fig. 6C).

Finally, we verified whether the changes in ERD and ERS occurring during the task were related to the changes in resting state over the same electrodes. We thus correlated the change in ERD and ERS in the three ROIs described previously with the RS change in the corresponding electrodes. No correlations were found with any electrodes in the three ROIs in either combined or single group analyses.

## Discussion

In this study, we examined for the first time the evolution of EEG oscillatory beta activity in electrodes over sensorimotor and frontal ROIs during a 40‐minute motor practice in patients with PD and in healthy age‐matched controls. We found that movement‐related modulation of beta power over the three ROIs changed significantly during practice in control subjects. In addition, in the controls, values of beta power recorded at rest, in the spontaneous EEG, substantially increased after practice in electrodes over frontal and left areas. In patients with PD, beta power at rest was higher than in controls and practice‐induced changes at rest, like those during movements, were markedly reduced compared to the control group.

### Beta movement‐related modulation is reduced in PD

Similar to other motor tasks (Alegre et al. 2004, 2006; Lim et al. 2006; Perfetti et al. 2011; Formaggio et al. 2015), our reaching task produced a pattern of beta modulation characterized by a power decrease during movement, or ERD, followed by an increase at the end of the movement, or ERS. This pattern was well‐defined in the three selected ROIs (Fig. 1) and in both groups. However, it was reduced in patients with PD compared to controls (see Fig. 4), in agreement with previous EEG and MEG studies that reported significant reductions of beta ERS accompanied, in some cases, by ERD reductions in PD (Cassidy et al. 2002; Delval et al. 2006; Kuhn et al. 2006; Lim et al. 2006; Hammond et al. 2007; Degardin et al. 2009; Dejean et al. 2009; Heinrichs‐Graham et al. 2014). What might be the reasons of the decreased movement‐related beta modulation in PD? While the precise mechanisms are not clear, associations with dopaminergic deficiency have been proposed. Indeed, levodopa administration can increase beta modulation in PD although it cannot restore it to the levels of healthy controls (Degardin et al. 2009). Since they were obtained during optimal pharmacological treatment, our results further suggest that ERD/ERS abnormalities in PD cannot be solely explained by dopaminergic deficiency. Other studies have suggested that decreased beta modulation is linked to the severity of motor signs, and in particular to akinesia (Labyt et al. 2003). However, in healthy subjects, the amplitudes of beta ERD and ERS seem to be rather insensitive to speed, force and movement type (Stancak and Pfurtscheller 1995, 1996; Stancak et al. 1997; Pistohl et al. 2012; Kilavik et al. 2013), suggesting that beta movement‐related modulation does not directly underlie the explicit coding of specific movement characteristics. Rather, as suggested by previous studies (Shimazu et al. 1999; Cassim et al. 2000), it may support a more global function, such as the regulation of sensory and motor interactions during different phases of movement planning and execution. This ability to filter incoming sensory information during movement has been sometimes referred to as “movement‐related gating”, a particular case of the more general phenomenon of “sensory gating” (Brown et al. 2015). In this context, beta ERD would reflect, concomitantly, activation of the motor areas and attenuation of the sensory afferences during movement; beta ERS instead, would reflect postmovement reactivation of somatosensory areas that, in turn, would induce inhibition or idle state of the motor areas. Along this line, there is evidence suggesting that modulation of beta power could be related to the amplitude of somatosensory evoked potentials components representing sensorimotor integration (Rossi et al. 2002; Cebolla and Cheron 2015). Such early components are suppressed during active, passive and observed movements (Abbruzzese et al.^1981^; Rushton et al. 1981 Brown et al. 2015). Thus, in the case of sensory or sensorimotor integration deficits, one should expect a dysregulation of such balance and, thus, an alteration of beta ERS and ERD patterns. Patients with PD often exhibit a variety of somatosensory abnormalities that can be subtle or blatantly present (Conte et al. 2013). Therefore, we speculate that reduced beta modulation during movement in PD shown in this and previous studies (Delval et al. 2006; Lim et al. 2006; Heinrichs‐Graham et al. 2014) could be related to a dysfunctional processing of somatosensory information. The importance of sensory afferences and their attenuation during motor performance, or “sensory gating”, has also been revisited by Friston and colleagues in a general scheme that brings together attention, motor preparation, proprioception as well as dopaminergic function (Friston et al. 2012; Brown et al. 2013). Indeed, further studies are needed to prove this scenario correct, to provide a direct link between PD sensory deficits and beta abnormalities and, thus, to address its specific mechanisms.

### Practice induces changes in movement‐related beta modulation

One of the major findings of this study is that in controls, beta ERS over the three ROIs increased significantly during practice; further, these practice‐related changes were considerably smaller in PD. This is the first report about changes of beta modulation occurring during repetitions of a simple motor task. A very limited number of EEG studies in control subjects have reported some changes that were invariably associated with performance improvement. Specifically, event‐related coherence increased with familiarity in motor tasks (Serrien and Brown 2003; Lange et al. 2006); event‐related potential amplitude increased with better accuracy in a visuomotor task (Staines et al. 2002); frontal theta and gamma power decreased with increase in automaticity in a mirror‐drawing task (Wong et al. 2014). Also, we have previously found that alpha and theta modulation changed during the learning of a visual sequence (Moisello et al. 2013). Interestingly, the changes in movement‐related beta modulation reported in this study were not clearly associated with improved performance: although movement speed increased in the 40‐minute practice in both groups, we did not find significant correlation between ERS beta changes and the decrease in movement time, in agreement with the results of recent studies (Tan et al. 2014). Indeed, this negative result could be explained in terms of groups’ size or the possible existence of a nonlinear correlation. However, comparison between movements of similar speed in the first and last blocks demonstrated significantly greater ERS values for movements performed in the last block, thus suggesting that speed improvement or kinematic optimization is not a major determinant of the changes of beta modulation during the movements. What could then drive the increase in beta ERS during motor practice in control subjects? First, as discussed in the previous paragraphs, in the context of “sensory gating”, the increased beta ERS rebound we found during the 40‐min task in control subjects might be interpreted as a more efficient switch from proprioceptive blockade to its reactivation (from ERD to ERS) induced by practice. Second, the fact that practice‐related beta ERS and ERD changes were prominent in the frontal ROI further supports the notion of involvement and optimization of attentional mechanisms, although in the absence of a clear behavioral correlate. Third, the reduced practice‐related beta changes in PD might be due to the altered sensory processing coupled with inefficiency of dopaminergic mechanisms. Also, in patients with PD, the aberrantly high beta power at rest could have played a role in preventing a further increase in movement‐related beta modulation in a sort of a “range effect”.

An important caveat for the interpretation of the changes in ERD and ERS is that such changes could be a reflection of the changes in beta power at rest that we found after the task. However, several facts indicate that this is an unlikely explanation. In fact, none of the ERD or ERS changes correlated with the change in resting power in any of the ROIs. Most importantly, similarly to ERS and ERD, the ERS/ERD peak‐to‐peak amplitude, an index of beta modulation that is independent from the mean power (see Supplemental Information), significantly increased with practice in the control group in the three ROIs. These changes in modulation “depth” were not significantly correlated with changes in either mean power during the movement or power during resting state.

### In PD beta power at rest is higher and does not change with practice

The finding of abnormally higher beta power at rest in PD confirms the results of previous studies showing higher power either in all or selected bands (Tanaka et al. 2000; Bosboom et al. 2006; Stoffers et al. 2007; Moazami‐Goudarzi et al. 2008). Also in agreement with other studies (Levy et al. 2002; Priori et al. 2004; Sharott et al. 2005; Kuhn et al. 2006; Mallet et al. 2008; Giannicola et al. 2010; Pollok et al. 2012; Brown et al. 2013; Tan et al. 2013), we found that the greater the beta power, the longer the disease duration and also the more severe the UPDRS III scores. Such power increase in PD has been interpreted as consequence of a general thalamocortical dysrhythmia (Llinas et al. 1999; Moazami‐Goudarzi et al. 2008). Another interpretation relies on the results of most recent studies with theta burst stimulation protocols. They generally showed an association between increases of beta power and decreases in cortical excitability (Hsu et al. 2011; Noh et al. 2012; McAllister et al. 2013). Evidence from both animal and human studies that high beta power reflects high GABA levels (Jensen et al. 2005; Roopun et al. 2006; Yamawaki et al. 2008; Hall et al. 2010, 2011; Muthukumaraswamy et al. 2012; Rossiter et al. 2014) further connects the increases in beta power to increases of inhibitory processes as well as to decreases of cortical excitability. Therefore, based upon these considerations, it is likely that the higher levels of beta power in PD are the expression of decreased cortical excitability, a finding that has been confirmed by electrophysiological and behavioral studies in PD ((Bedard and Sanes 2011; Kishore et al. 2012; Marinelli et al. 2009; Morgante et al. 2006), for a review see also (Koch 2013) and references therein). Along this line of reasoning, the significant posttask beta power increase in controls can be interpreted as a reduction in cortical excitability resulting from protracted use. This phenomenon is akin to the “occlusion” of LTP‐like plasticity, an event that occurs after motor practice and that has been measured with decreased response to paired associative TMS paradigms in the motor cortex of healthy subjects (Ziemann et al. 2004; Stefan et al. 2006; Cantarero et al. 2013). Occlusion of LTP‐like plasticity has been explained by saturation of the synaptic modification range, a situation that prevents the occurrence of subsequent LTP‐like plasticity. If saturation of plasticity‐related mechanisms is the cause of posttask increased beta activity, rest, and sleep should be able to restore beta levels to the original levels, as we have found in preliminary studies (C. Moisello and M. Felice Ghilardi, unpubl. data). These practice‐related changes at rest did not correlate with those during performance, implying that the two sets of changes must reflect different phenomena. Finally, the failure in patients to display significant posttask increase could be ascribed to a to an already “over‐inhibited” state or an occlusion of LTP‐like plasticity reflected by high beta power levels.

## Conclusions

This is the first study showing that beta oscillations increased during and after motor practice in healthy controls. Such changes are reduced in PD. These changes were not linked to changes in kinematic variables. We speculate that, on the one hand, the increase in beta modulation during movement might be expression of a “refinement” of the sensory gating phenomenon. In PD, this might be reduced because of somatosensory abnormalities that are subtle but often present in PD. On the other hand, based on the result of previous studies combining EEG and LTP‐like protocols in humans, the posttask increase in the resting state EEG might reflect saturation of LTP‐like plasticity caused by practice of the task. In patients with PD, beta power changes might be less evident because of the higher beta power, a reflection of abnormal plasticity and occlusion of LTP‐like plasticity.

## Conflict of Interest

None declared.

## Supporting information


**Figure S1**. Group average of normalized movement area across Blocks in patients with PD (filled circles) and controls (empty circles) during the 40‐minute reaching task.
**Figure S2**. Group average of within‐block movement variability (expressed standard deviation) in patients with PD (filled circles) and controls (empty circles) for the indicated behavioral measures.
**Figure S3**. Group average of beta ERD–ERS peak‐to‐peak amplitude indicating beta modulation depth (MD) across time bins in patients with PD (filled circles) and controls (empty circles) during the 40‐minute reaching task.
