# Supplementary material for: Practice changes beta power at rest and its modulation during movement in healthy subjects but not in patients with Parkinson's disease
Source: Brain Behav. 2015 Sep 23;5(10):e00374. doi: 10.1002/brb3.374 (PMC4614055; doi:10.1002/brb3.374)
Supplement: Supplementary file 1 — Figure S1. Group average of normalized movement area across Blocks in patients with PD (filled circles) and controls (empty circles) during the 40‐minute reaching task. Figure S2. Group average of within‐block movement variability (expressed standard deviation) in patients with PD (filled circles) and controls (empty circles) for the indicated behavioral measures. Figure S3. Group average of beta ERD–ERS peak‐to‐peak amplitude indicating beta modulation depth (MD) across time bins in patients with PD (filled circles) and controls (empty circles) during the 40‐minute reaching task. [file BRB3-5-0f-s001.doc]

# Supplemental Material.

**Results – Timing of ERD/ERS**

We verified that the timing of ERD and ERS was similar between groups (ERD, controls: 0.23 ± 0.17 s after movement onset; PD: 0.24 ± 0.18 s; unpaired t-test: p=0.71; ERS, controls: 1.09 ± 0.12 s; PD: 1.12 ± 1.6 s; unpaired t-test: p=0.36).

**Results – Accuracy**

While the main behavioral outcomes are reported in the main text, in this supplemental material we report the statistical analysis of an index that best reflects movement accuracy, namely the “normalized movement area”. The normalized area is computed as the area enclosed in the movement trajectory (both forward and backward movement), divided by the squared out-path length (see also Moisello et al. 2008). As such, it’s an a-dimensional index that reflects the level of ‘overlapping’ of out-and-back strokes, as specified in the task’s instruction. We performed the same mixed model ANOVA described in the Methods section of the main manuscript.

As reported in Figure 1S, the mean normalized area did not differ between groups (Effect of Group: F(1, 29)=1.06, p=0.31) and significantly decreased with practice in both groups (Effect of Practice: F(9, 261)=3.98; p=0.0008; Group x Practice: F(9,261)=0.94, p=0.49), thus indicating a improvement in accuracy with task performance.


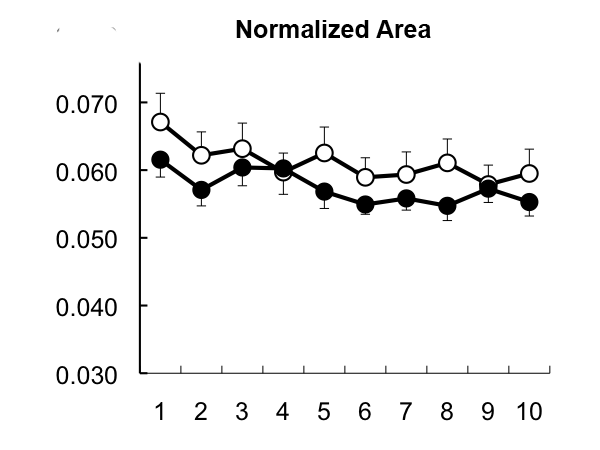


**Figure S1**. Group average of normalized movement area across Blocks in patients with PD (filled circles) and controls (empty circles) during the forty-minute reaching task. Bars represent standard errors.

**Results – Variability of Movement parameters**

We further verified the presence of an effect of practice on the within-block variability of the movement parameters (reaction time, movement time, extent and normalized area). Reaction time variability was significantly higher in PD compared to controls (Group: F(1, 29)=15.06, p=0.0006), but did not change with practice in either group (Practice: F(9, 261)=0.36; p=0.95; Group x Practice: F(9,261)=1.30, p=0.23). Movement time variability was slightly higher in PD (Group: F(1, 29)=2.89, p=0.09), and decreased similarly with Practice in both groups (Practice: F(9, 261)=4.1; p=0.00006; Group x Practice: F(9,261)=0.63, p=0.76). No significant effect was found for movement extent (p always > 0.13), while a mild effect of practice was present in the variability of Normalized Area (F(9, 261)=2.93; p=0.02).


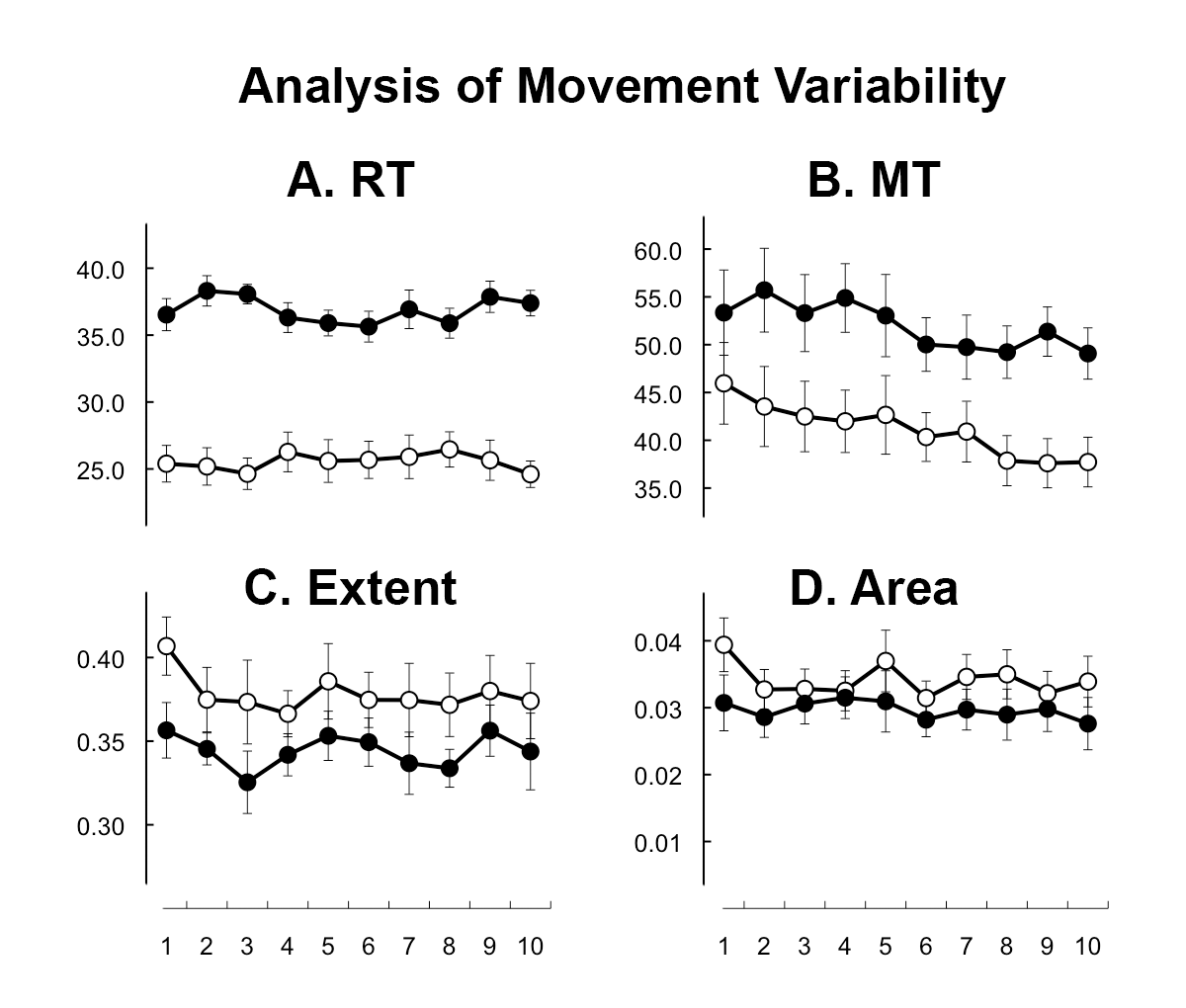


**Figure S2**. Group average of within-block movement variability (expressed standard deviation) in patients with PD (filled circles) and controls (empty circles) for the indicated behavioral measures. Bars represent standard errors.

**Results – Beta peak-to-peak amplitude**

To verify whether changes in ERD and ERS were just a reflection of the change of mean power that we observed in the resting state, we performed additional analyses. Specifically, in each subject, we computed an index of modulation depth, measured as the beta ERD - ERS peak-to-peak amplitude (see also te Woerd et al. 2014). By definition, such index is independent of the change of mean power. We then performed the same mixed model ANOVA described in the Methods section of the main manuscript.

In general, the results obtained with this analysis are similar to those obtained with ERS analyses described in the main manuscript.

*Left ROI (Figure 3S-A)*. On average, the peak-to-peak amplitude was significantly higher in the control compared to the PD group (F(1, 29)=6.44, p=0.02). Importantly, it increased significantly during practice (F(9, 261)=8.66, p<0.0001), with a significant Group x Practice interaction (F(9, 261)=2.82, p=0.003), indicating that such increase was more pronounced in the controls. Indeed, post-hoc tests showed a significant increase only in the controls, starting from Block4 (compared to Block1: p=0.001 and always significant thereafter). In the PD group, none of the comparisons yielded significant results (p always >0.9).

*Right ROI (Figure 3S-B)*. Peak-to-peak amplitude was similar in the two groups (F(1, 29)=1.31, p=0.26) and significantly increased with practice (F(9, 261)=5.34, p<0.0001), with a significant Practice x Group effect (F(9, 261)=2.33, p<0.02). This practice-related increase was significant in the control group (from Block5: p always <0.01), but not in the PD group (p always >0.9).

*Frontal ROI (Figure 3S-C).* Peak-to-peak amplitude was significantly different in the two groups (F(1, 29)=8.56, p=0.007) and increased during practice in both group (Practice: F(9, 261)=3.88, p=0.001) with a trend for Practice x Group interaction (F(9, 261)=1.81, p=0.07). Even for this ROI, changes with practice were significant only in the controls, starting from Block5 (p=0.02).

Overall, the results of these analyses supports the use of peak-to-peak amplitude as a valuable index to capture beta dynamics during a motor task, especially in the context of fast repeated movements, where an inter-movement baseline might not be too reliable.

Importantly, the peak-to-peak amplitude changes over the three ROIs were not significantly correlated with the change in resting state power (r<0.18, p>0.33), thus suggesting that the change in beta modulation occurring with practice cannot be ascribed to the shift in resting state power.


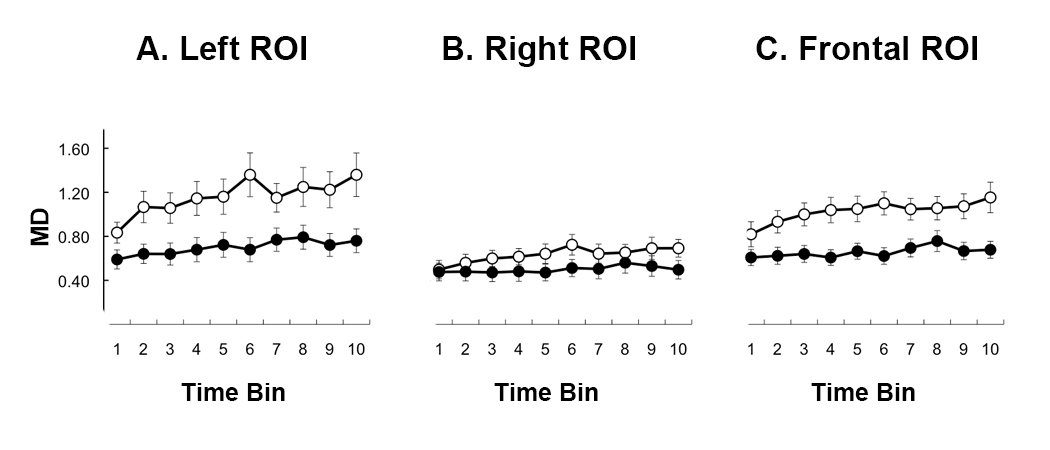


**Figure S3**. Group average of beta ERD - ERS peak-to-peak amplitude indicating beta modulation depth (MD) across time bins in patients with PD (filled circles) and controls (empty circles) during the forty-minute reaching task. Bars represent standard errors. A-B-C. Time course of beta MD values for the Left, Right and Frontal ROIs.
